# Characteristics of Korean students advised to seek psychiatric treatment before death by suicide

**DOI:** 10.3389/fpsyt.2022.950514

**Published:** 2022-09-06

**Authors:** Hee Jin Kim, Yong-Sil Kweon, Hyun Ju Hong

**Affiliations:** ^1^Department of Psychiatry, Hallym University Sacred Heart Hospital, Anyang, South Korea; ^2^Department of Psychiatry, The Catholic University of Korea College of Medicine, Seoul, South Korea; ^3^Suicide and School Mental Health Institute, Anyang, South Korea

**Keywords:** student, suicide, psychiatric treatment, school, teacher

## Abstract

**Introduction:**

Suicide is the leading cause of death among adolescents in Korea. Psychiatric disorders are well-known risk factors for suicide, but the proportion of children and adolescents who died by suicide and who had received psychiatric treatment is low. This study aims to examine how many school students who died by suicide were advised by their school to seek psychiatric treatment before their death and to characterize their clinical characteristics.

**Methods:**

We analyzed data collected by the Ministry of Education of Korea for all students who died by suicide between 2016 and 2020. Students were grouped according to whether or not they were advised to seek psychiatric treatment by their school-based on mental health screening and teachers’ judgments. Sociodemographic characteristics (sex, educational stage, family structure, and socioeconomic status), suicide-related characteristics (place of suicide, suicide method, suicide note, previous self-harm, and previous suicide attempt), emotional and behavioral status, school life and personal, and family problems were compared between the two groups.

**Results:**

Analysis was conducted for 544 students, 110 (20.2%) of whom were advised to seek psychiatric treatment by their school before their death. This group had a higher proportion of girls; poorer attendance; higher frequency of depression, anxiety, impulsivity, and social problems; personal problems (appearance, friend-related, and mental and physical health problems); family problems (mental health problems of family, bad relationship with parents, and conflict of parents); and higher incidence of self-harm or suicide attempts (*P* < 0.001) than the other group.

**Conclusion:**

Teachers seem to advise psychiatric treatment when mental health problems are revealed at school. It showed distinctive clinical characteristics between the two groups. Preventing suicide among students requires the attention and effort not only of schools, but also of families, communities, and mental health professionals.

## Introduction

Approximately 800,000 people worldwide die by suicide every year. This translates to a rate of one suicide death every 40 s (1).^1^ According to the World Health Organization, suicide is an emerging major mental health problem and the fourth leading cause of death among people aged 15–19 years in 2021 (2).^2^ Suicide is the leading cause of death in Korean teenagers (3).^3^ It is heterogeneous, with various risk and protective factors, and its characteristics differ between adolescents and adults (4). Psychiatric conditions such as depression, schizophrenia, and bipolar disorder are well-known risk factors for suicide (5). According to a psychological autopsy study of adolescents, 90% of suicidal adolescents have psychiatric disorders, with the most common being affective disorder (6, 7). However, less than a third of adolescents who died by suicide received any psychiatric treatment (6–8).

Effective treatment, including pharmacotherapy and psychotherapy for psychiatric disorders, may reduce suicide rates. Cognitive therapy halved the rate of retry attempts in suicide attempters compared to those who received conventional treatment (9). Suicidal ideation was also reduced with interpersonal psychotherapy, cognitive behavioral therapy, and dialectical behavioral therapy (10). Education of primary care physicians about mental illness and suicide risk can contribute to a reduction in suicide rates. A Swedish study found a decrease in the suicide rate 3 years after educating primary care physicians on the diagnosis and treatment of depression (11). Depression management education programs for primary care physicians reduced rates of suicide attempts using high-risk methods such as hanging or jumping in Germany (12) and overall suicide rates in Hungary (13). A study of Eastern European countries found that having a high number of doctors per capita was associated with an increased awareness of depression and low suicide rates (14). In particular, an increase in the availability of psychiatrists was associated with decreased suicide rates (14, 15). In addition, a systematic review of randomized trials found that lithium administration reduced the risk of death and suicide by 60% in people with mood disorders (16). In the United States, research shows an inverse association between the number of psychiatric wards and suicide rates (17); also, the diagnosis and treatment of depression might have improved, with a resulting decrease in suicide rates (18). In Europe, the use of antidepressants is one of the most important interventions to reduce the suicide rate (19–21). Prescription of selective serotonin reuptake inhibitors is associated with decreased early adolescent suicide (22). Also, there are decreased suicide attempts among adolescents treated with antidepressants for 6 months (23).

Children and adolescents spend a lot of time at school and school based-suicide prevention policies are commonly implemented for preventing suicide in this demographic in many countries. Sign of Suicide (24), Lifeline (25), and Question, Persuade, and Refer (26) are well-known education programs for suicide prevention. In addition, a reduction in the risk of suicide has been reported after the implementation of the Youth Aware of Mental Health School Program in Saving and Empowering Young Lives in Europe, a school-based suicide prevention project in the European Union (27). In Korea, a school-based mental health screening program using the Students’ Emotional and Behavioral Screening Test (SEBT) has been implemented since 2012 for all schools across the country. High risk students for mental health issues are identified based on their test score. These students are advised to visit out-of-school mental health service providers, including psychiatric clinics. Recently, the national school-based mental health policy and the management of students at high risk for mental health were strengthened (28).^4^

In a study regarding sources of referral of child and adolescent psychiatric outpatients in Korea, 23.7% of cases involving suicidal or self-harming behaviors came from schools, which was much higher than referrals from other channels (29). However, since teachers meet students in the limited environment of a school, there are limits to integrated evaluation of a student’s problems. Furthermore, teachers are more sensitive to externalizing than internalizing symptoms (30, 31). Also, teachers’ perception of mental illness may be different from that of a psychiatrist. For example, when presented with a case of attention deficit hyperactivity disorder, only 38% of teachers correctly identified the disorder, and they preferred to use a strategy that can be implemented at school rather than drug treatment (32). According to previous studies, 7–36% of children and adolescents who died by suicide had a history of psychiatric treatment (6–8), but it is unknown how many of them had been advised by the school to seek psychiatric treatment. This study aims to examine how many students who died by suicide were advised by their school to seek psychiatric treatment before their death, and to identify the clinical characteristics of students who were advised to seek psychiatric treatment by the school, based on Korean data for 2016–2020.

## Materials and methods

### Database

We analyzed the 2016–2020 Student Suicide Reports, an official database of all school students who died by suicide collected by the Ministry of Education of Korea. When students die by suicide, their teachers submit a report to the Ministry of Education. Student Suicide Reports, which include teachers’ observations, parental reports regarding the circumstances of death, and information collected by the school as official education records, are collated as part of the national student suicide prevention policy. The collected data contains the student’s sociodemographic as well as suicide-related information, individual and family characteristics, information about school life and interpersonal relationships, and about suicide prevention and postvention at school. We included data for students from the second grade of elementary school to the third year of high school, corresponding to ages 7–18 years.

This study was approved by the Institutional Review Board of Hallym University Sacred Heart Hospital.

### Analyzed variables

Students’ psychiatric treatment recommended by the school was classified according to whether they were identified as high risk based on their SEBT scores or whether the teacher made the decision based on other factors. The SEBT is administered annually to all first and fourth graders at elementary schools and first-year students at middle and high schools in Korea, and approximately 3–5% of students are classified as high risk (33). A final decision is made after the teacher interviews students who have exceeded the cut-off score in self-assessment (middle and high school students) or parental evaluation (elementary school students). More detailed information on the process of SEBT and the contents and reliability of the measurements published in the previous paper (34). When a student is identified as high risk based on the SEBT, the school provides in-school counseling and also advises parents to take their children to out-of-school mental health specialists which include public mental health centers, youth counseling centers, psychiatric clinics and etc. Of the 1,931,955 students, 3.2% were screened as high risk, and 8.2% of high risk students were recommended for psychiatric treatment in 2016 (33). Of the 654 students who died by suicide in 2015–2020, 110 were advised to undergo psychiatric treatment, 42 through SEBT, 99 through teacher judgment, and 31 through both SEBT and teacher judgment.

In this study, the sociodemographic characteristics (e.g., sex, educational stage, family structure, and socioeconomic status), suicide-related characteristics (e.g., place of suicide, suicide method, suicide note, previous self-harm, and previous suicide attempt), emotional and behavioral status (e.g., depression, anxiety, impulsivity, and social problems), and school life of the two groups were compared. The teacher-rated Strengths and Difficulties Questionnaire (SDQ) was used to evaluate the emotional and behavioral status of students. The teacher-rated SDQ consists of prosocial behavior (Cronbach’s α = 0.873), hyperactivity/inattention (Cronbach’s α = 0.793), peer relationship problems (Cronbach’s α = 0.770), emotional symptoms (Cronbach’s α = 0.681), conduct problems (Cronbach’s α = 0.638), and total difficulties score (Cronbach’s α = 0.837). School-related factors included school attendance, academic achievement, relationships with teachers, and conduct problems. Personal and family-related problems were assessed with yes-or-no questions and include items on physical and mental health problems, appearance, friend-related problems, addictions, relationships with parents, conflicts with parents, economic problems, and violence. The “appearance problem” meant inferiority in appearance and “smartphone addiction” was evaluated through the subjective judgment of teachers, not by objective measures.

### Statistics

Independent sample *t*-tests were performed for continuous variables (e.g., SDQ). Sociodemographic characteristics, suicide-related characteristics, emotional and behavioral status, school-related factors, and personal and family-related problems (categorical variables) were compared between the two groups using frequency analysis, Chi-squared test, and Fisher’s exact test. The statistical significance level was set to 0.05 for univariate analyses and adjustment for multiple tests was also made by dividing the *P*-value by the number of comparisons (Bonferroni correction). Further analysis was performed using multiple logistic regression to assess the independent contribution of variables associated with students advised to seek psychiatric treatment before death by suicide. All statistically significant variables adjusting multiple comparison (*P* < 0.0014) were entered into a multiple logistic regression model. Statistical Package for the Social Sciences ver. 27 (SPSS; IBM Corp., Armonk, NY, United States) was used.

## Results

We retrieved data for 654 students who died by suicide. After exclusion of records lacking psychiatric treatment-related information, 544 students were included in the analysis. Among the 106 students (19.5% of 544) in the high risk group identified using the SEBT, 42 students (39.6% of 106) were advised to seek psychiatric treatment. Of the 191 students (35.1% of 544) who were identified as high risk by teachers, 99 (51.8% of 191) were advised to seek psychiatric treatment. Of the 141 students who were advised to seek psychiatric treatment, 31 cases were advised to seek psychiatric treatment *via* both channels. In total, 110 students (20.2%) were advised to seek psychiatric treatment (AP-group) and 434 (79.8%) were not advised to seek psychiatric treatment (NAP-group).

### Sociodemographic characteristics

Of the 544 records, 288 (52.9%) were of boys and 256 (47.1%) were of girls. Of these, 36 (32.7%) boys were advised to get psychiatric help, whereas 74 (67.3%) girls were given this advice. After adjusting for multiple comparisons, there are more girls in the AP group (χ^2^ = 22.613, *P* < 0.001) (Table 1).

**TABLE 1:** Sociodemographic characteristics.

		NAP (*N* = 434)	AP (*N* = 110)	χ^2^	*P*
	*N* (%*^a^* of total responses)	*N* (%*^a^* of NAP)	*N* (%*^a^* of AP)		
**Sex**					
Girls	256 (47.1)	182 (41.9)	74 (67.3)	22.613*	<0.001
Boys	288 (52.9)	252 (58.1)	36 (32.7)		
**Educational stage**					
Elementary school	17 (3.1)	14 (3.2)	3 (2.7)	0.164	0.086
Middle school	170 (31.3)	134 (30.9)	36 (32.7)		
High school	357 (65.6)	286 (65.9)	71 (64.5)		
**Family structure**					
Both parents	384 (72.9)	314 (74.4)	70 (66.7)	2.548	0.11
Single parents or others	143 (27.1)	108 (25.6)	35 (33.4)		
**Socioeconomic status**					
Lower	109 (24.1)	77 (21.4)	32 (34.0)	6.479	0.039
Middle	298 (65.8)	244 (68.0)	54 (57.4)		
Upper	46 (10.2)	38 (10.6)	8 (8.5)		

NAP, not advised to seek psychiatric treatment; AP, advised to seek psychiatric treatment.

*P < 0.0014, statistically significant after Bonferroni correction for the multiple testing.

^a^The percentage specified is the column percentage.

### Characteristics related to suicide

The AP group were more likely to have self-harmed (53.1 versus 3.9%; χ^2^ = 150.32, *P* < 0.001) or to have previously attempted suicide (38.1 versus 3.5%; χ^2^ = 87.947, *P* < 0.001) than the NAP group. The most common suicide location was the home, accounting for more than 50% in both groups (NAP 63.7%, AP 67.6%), and jumping or drowning were the most frequent suicide methods (NAP 70.6%, AP 69.4%). The rate of presence of a suicide note was similar in both groups at around 50%. (NAP 50.8%, AP 51.2%) After adjusting for multiple comparisons, these variables still showed statistical significance (Table 2).

**TABLE 2:** Characteristics related to suicide.

		NAP (*N* = 434)	AP (*N* = 110)	χ^2^	*P*
	*N* (%*^a^* of total responses)	*N* (%*^a^* of NAP)	*N* (%*^a^* of AP)		
**Place of suicide**					
Home	347 (64.5)	274 (63.7)	73 (67.6)	3.445	0.328
School	12 (2.2)	12 (2.8)	0 (0.0)		
Public places	143 (26.6)	116 (27.0)	27 (25.0)		
Others	36 (6.7)	28 (6.5)	8 (7.4)		
**Suicide method**					
Hanging	140 (26.1)	115 (26.9)	25 (23.1)	6.165	0.45
Jumping or drowning	377 (70.3)	302 (70.6)	75 (69.4)		
Others	19 (3.5)	11 (2.6)	8 (7.4)		
**Suicide note (+)**	238 (50.9)	194 (50.8)	44 (51.2)	0.004	0.95
**Previous self-harm (+)**	65 (14.3)	14 (3.9)	51 (53.1)	150.32*	<0.001
**Previous suicide attempt (+)**	44 (10.3)	12 (3.5)	32 (38.1)	87.947*	<0.001

NAP, not advised to seek psychiatric treatment; AP, advised to seek psychiatric treatment.

*P < 0.0014, statistically significant after Bonferroni correction for the multiple testing.

^a^The percentage specified is the column percentage.

### Behavioral and emotional problems

Regarding the characteristics of emotional behavior, the AP group had higher rates of depression, anxiety, impulsivity, and social problems (*P* < 0.001). In SDQ, there were significant differences across all components. The prosocial behavior score (*P* < 0.001) was higher in the NAP group, but the AP group scored higher in all problem areas: emotional symptoms (*P* < 0.001), conduct problems (*P* = 0.004), hyperactivity/inattention (*P* < 0.001), and peer relationship problems (*P* < 0.001). After adjusting for multiple comparisons, these variables showed statistical significance except conduct problems (Table 3).

**TABLE 3:** Behavioral and emotional problems.

		NAP (*N* = 434)	AP (*N* = 110)	χ^2^	*P*
	*N* (%*^a^* of total responses)	*N* (%*^a^* of NAP)	*N* (%*^a^* of AP)		
Depression	148 (30.0)	63 (16.2)	85 (80.2)	162.267*	<0.001
Anxiety	70 (14.2)	20 (5.2)	50 (47.2)	120.841*	<0.001
Impulsivity	62 (12.6)	32 (8.2)	30 (28.3)	30.508*	<0.001
Social problems	56 (11.3)	28 (7.2)	28 (26.4)	30.531*	<0.001

**SDQ**	**Mean of total responses (SD)**	**Mean (SD)**	**Mean (SD)**	***T***	***P***

Emotional symptoms	2.31 (2.27)	1.64 (1.75)	4.74 (2.28)	−12.16*	<0.001
Conduct problems	1.27 (1.42)	1.16 (1.37)	1.68 (1.53)	−2.963	0.004
Hyperactivity/inattention	2.59 (2.24)	2.31 (2.11)	3.61 (2.41)	−4.727*	<0.001
Peer relationship problems	2.12 (1.89)	1.78 (1.70)	3.35 (2.03)	−6.831*	<0.001
Prosocial behavior	5.96 (2.61)	6.21 (2.49)	5.06 (2.83)	3.768*	<0.001

NAP, not advised to seek psychiatric treatment; AP, advised to seek psychiatric treatment; SDQ, Strengths and Difficulties Questionnaire; SD, standard deviation.

*P < 0.0014, statistically significant after Bonferroni correction for the multiple testing.

^a^The percentage specified is the column percentage.

### School-related factors

Regarding school-related factors, there was no significant difference between the prevalence of school bullying perpetrators and victims in either group. The AP group was more likely to have poor attendance (60.9%), perform below par academically (55.1%), has poor relationships with teachers (16.7%), smoke (16.7%), and drink (7.0%). After adjusting for multiple comparisons, school attendance was significantly more frequent in the AP group (Table 4).

**TABLE 4:** School-related factors.

		NAP (*N* = 434)	AP (*N* = 110)	χ^2^	*P*
	*N* (%*^a^* of total responses)	*N* (%*^a^* of NAP)	*N* (%*^a^* of AP)		
**School attendance**					
Good	376 (70.0)	333 (78.0)	43 (39.1)	63.033*	<0.001
Poor	161 (30.0)	94 (22.0)	67 (60.9)		
**Academic achievement**					
Above average	113 (21.7)	97 (23.4)	16 (15.0)	11.86	0.003
Average	196 (37.6)	164 (39.6)	32 (29.9)		
Below average	212 (40.7)	153 (37.0)	59 (55.1)		
**Relationship with teachers**					
Amicable	405 (91.2)	325 (93.4)	80 (83.3)	9.499	0.002
Discordant	39 (8.8)	23 (6.6)	16 (16.7)		
**Conduct problems**					
Smoking	50 (9.5)	65 (7.7)	18 (16.7)	8.102	0.004
Drinking	37 (7.0)	23 (5.5)	14 (13.0)	7.305	0.007
School bullying perpetrator	11 (2.1)	6 (1.4)	5 (4.6)	4.277	0.054
School bullying victim	1 (1.2)	1 (1.4)	0 (0.0)	0.202	1.000

NAP, not advised to seek psychiatric treatment; AP, advised to seek psychiatric treatment.

*P < 0.0014, statistically significant after Bonferroni correction for the multiple testing.

^a^The percentage specified is the column percentage.

### Personal and family-related problems

For both personal and family-related problems, there were significant differences between the two groups in most variables. Physical problems, mental health problems, friend-related problems, appearance related problems, mental health problems in the family, bad relationship with parents, and conflict of parents were more frequent in the AP group even after adjusting for multiple comparisons (*P* < 0.001) (Table 5).

**TABLE 5:** Personal and family-related problems.

		NAP (*N* = 434)	AP (*N* = 110)	χ^2^	*P*
	*N* (%*^a^* of total responses)	*N* (%*^a^* of NAP)	*N* (%*^a^* of AP)		
**Personal problems**					
Physical health problems	44 (9.4)	24 (6.6)	20 (19.0)	14.868*	<0.001
Mental health problems	113 (24.1)	28 (7.7)	85 (81.0)	239.15*	<0.001
Friend-related problems	89 (18.5)	54 (14.0)	35 (36.5)	25.642*	<0.001
Appearance related	37 (7.9)	19 (5.2)	18 (17.1)	15.942*	<0.001
Smartphone addiction	28 (6.4)	18 (5.0)	10 (12.2)	5.752	0.024
**Family-related problems**					
Physical health problems in the family	14 (3.3)	7 (2.1)	7 (7.6)	6.969	0.016
Mental health problems in the family	21 (4.9)	8 (2.4)	13 (14.1)	21.37*	<0.001
Bad relationship with parents	175 (42.2)	120 (37.0)	55 (60.4)	15.956*	<0.001
Conflict of parents	132 (30.8)	87 (25.9)	45 (48.9)	17.944*	<0.001
Violence	14 (3.3)	6 (1.8)	8 (8.7)	10.899	0.003
Economic problems	56 (13.1)	35 (10.4)	21 (22.8)	9.78	0.002

NAP, not advised to seek psychiatric treatment; AP, advised to seek psychiatric treatment.

*P < 0.0014, statistically significant after Bonferroni correction for the multiple testing.

^a^The percentage specified is the column percentage.

### Multiple logistic regression

Past history of suicide attempt under “Characteristics related to suicide” and physical health and mental health problems under “Personal problems” were independent factors related to receiving psychiatric treatment recommendations from the school among students died by suicide (Table 6). Other variables were not significant.

**TABLE 6:** Statistically significant variables of multiple logistic regression.

Variables	Coefficient	SE	OR	95% CI	*P*
Previous suicide attempt	3.984	1.470	53.739	3.015–957.863	0.007
Physical health problems	3.179	0.974	24.022	3.562–162.015	0.001
Mental health problems	3.758	0.890	42.853	7.493–245.059	<0.001

SE, standard error; OR, odds ratio; CI, confidence interval.

## Discussion

We analyzed a representative sample of the suicide data of all school students in Korea and found that approximately 20% of students who died by suicide in Korea were advised to seek psychiatric treatment by their school. Most students who received psychiatric treatment recommendations were female, had severe emotional and behavioral problems, had a high rate of history of suicide or self-harm, had a poor school attendance, and had negative peer and family relationships. In particular, the history of suicide attempts and mental or physical health problems were the variables that had the greatest influence on the teacher’s decision to recommend psychiatric treatment. In other words, when suicide risk factors such as conduct, depression, anxiety (35), difficult family or friend relationships (36, 37), and self-harm attempts (38) were confirmed by teachers at school, teachers were likely to advise psychiatric treatment. However, 80% of students who died by suicide, and whose emotional and behavioral problems were not apparent to the teachers, were not advised to seek psychiatric treatment.

According to a systematic review of adult psychological autopsy studies, approximately 90% of patients had mental illness (39). Furthermore, according to the psychological autopsy of suicide conducted on adults in Korea, it was estimated that 619 out of 698 included individuals who died by suicide (88.7%) had psychiatric disorders (40).^5^ A Chinese psychological autopsy study found that 63% of all suicides were related to mental illness (41), and it was recently found that antidepressant use is associated with a low suicide rate (42). Studies on suicide in children and adolescents reveal that mental illness is an important risk factor for suicide. In a psychological autopsy study in the United States, psychiatric disorders were diagnosed in more than 90% of cases (6), and in a Finnish study, the majority of participants had mental disorders (43). However, approximately, less than a third of adolescents who died by suicide received psychiatric treatment (6–8). According to a psychological autopsy study of adults who attempted suicide in Korea, 306 (43.8%) of 698 participants had received psychiatric treatment (236 outpatients and 70 hospitalized; see text footnote 5) (40). However, there have been no published studies on psychiatric treatment among Korean students who attempted suicide. According to the 2017 Adolescent Health Behavior Survey in Korea, approximately 2.6% of adolescents exhibited suicidal behavior for over 1 year, but only 17.9% of whom had received psychiatric treatment (44).^6^ In our study, 20% of students who died by suicide were advised through the school to seek psychiatric treatment. The proportion of cases in which teachers suspected psychiatric disorders among suicide students was much lower than the psychiatric disease rates in the existing literature; teachers seem to advise psychiatric treatment when emotional and behavioral problems are apparent at school. But the result does not imply that teachers are suboptimally aware of suicide-related psychopathologies of students. Most students who were not advised to seek psychiatric treatment in this study did not show apparent problems at school, so it may be difficult for even mental health professionals to recognize these students’ problems in school.

However, the proportion of 20% of students being advised to seek psychiatric treatment may not accurately reflect the rate at which such treatment was received. Because some students had already received or were receiving psychiatric treatment independent of school advisement, the actual rate of psychiatric treatment may be higher than the rate of being advised to seek psychiatric treatment. Considering Korea’s recent forceful school mental health policy, which includes intensive case management beyond simple screening, it seems likely that more than 20% of students were actually receiving psychiatric treatment. However, we have no information on whether such psychiatric treatment was continued until death. For effective suicide prevention, emotional and economic support of high risk suicide students and their families should be strengthened so that students with severe problems do not cease psychiatric treatment. It may also be helpful to expand family education on detecting depression, and to establish a social system that can detect and intervene early on against high risk families that practice abuse, neglect, and domestic violence.

In our study, 80% of students who died by suicide were not advised by their school to seek psychiatric treatment. They had relatively good attendance, good emotional and behavioral status, no family and friendship problems, and were less likely to have a history of self-harm or suicide attempts. This study suggests that even if children and adolescents have suicidal thoughts and mental illness, their school function may appear to be good. This emphasizes that a significant number of suicidal students may not reveal their psychological difficulties at school and may express it online, or to friends and family, but not to teachers. It is possible that a part of suicide death among adolescents is unrelated to detectable mental illness or persistent psychopathology, and it suggests the heterogeneity of adolescent suicide. According to the study on clustering of adolescent suicide of Korea, the silent type did not show obvious risk factors like mental illness (45). This finding has also been reported in other countries. In psychological autopsy studies, among students who died by suicide, there was a type of adolescent who functioned relatively well and did not exhibit any known risk factors of suicide or mental illness, making the suicide essentially unpredictable (46, 47). In a psychological autopsy study of adolescents in Israel, this unpredictable subpopulation of students who died by suicide, called the tragic narrative type, was not obvious up until just the suicide occurred; they showed low impulsivity and substance abuse rates, and had good academic performance (46). Furthermore in a United Kingdom study, one group of suicide students, appeared to be without apparent mental illness and functioning well, but tended to be overwhelmed by their unstable personality, high expectations for themselves, aspirations for perfection, and perceived failure (47). Even people without mental health issues (48),^7^ including students (38), often report that they will not actively seek treatment to avoid stigma even if they become mentally ill, and the characteristics of the students who died by suicide described here may have made them highly concerned about exposure to stigma and highly reluctant to ask for help (46, 49). Not much is known about the various types of adolescents who died by suicide, and further studies need to include various information as well as schools.

According to psychological autopsies, depression accounts for approximately 60% of affective disorders in suicide victims (43), leading to feelings of hopelessness and low energy, which may have made it even more difficult to seek help (50). To prevent suicide, children and adolescents should be encouraged to request help more readily when they are experiencing psychological difficulties, and teachers and parents need to respond more sensitively to the emotional state of children and adolescents.

In young adolescents, suicide risk signs or antecedent factors may not be as clearly apparent as in older adolescents (51). A significant proportion of students die by suicide in elementary school, compared with older youth, and they die from impulsive suicide attempts due to acute stress (52) rather than due to chronic mental health problems (53). In other words, because of the characteristics of young adolescents, emotional and behavioral problems are not always obvious at school (52, 53), making it more difficult for teachers to recognize suicide risk. Even in the case of the above tragic narrative type of adolescents, it may be difficult to recognize the risk of suicide through a single screening test because psychological symptoms and social functions may fluctuate within a short period of time immediately before suicide, and mental pain is not disclosed (46). In addition, it is rare for young people to suddenly disclose their emotional distress or suicidal thoughts and behaviors (54). Also, as suicide ideas are related to reduced requests for help (55), only one fifth of adolescents reveal that they have suicidal thoughts (54–56). Often not even mothers know about their children’s self-harm (57), suicide attempts and suicidal thoughts (58). To counter these difficulties, it is necessary to create a system that makes it easier for students with suicidal ideation to receive appropriate mental health services before they progress to suicide attempts.

According to the 2021 National Mental Health Awareness and Attitude Survey, many Koreans hesitate to seek psychiatric treatment because of the negative views of those around them (48).^8^ According to our study, teachers recommended to 22.1% (120/544) of students who showed emotional and behavioral problems at school that they should visit a mental health service institution rather than to seek psychiatric treatment. For school-based suicide prevention, education for teachers and students on the topic needs to be strengthened. This must include the characteristics of students who died by suicide, suicide warning signs, suicide risk and protective factors, encouraging help seeking behavior, and a general understanding of mental illness and psychiatric treatment. Efforts should be undertaken to reduce negative perception of psychiatric diseases and their treatment in society as a whole.

Our study had several limitations. First, student suicide reports were based on teacher’s reports collected by the school. Information on student’s internalized problems or family issues was limited. Second, it is likely that not all members of the group who were advised to seek psychiatric treatment received actual psychiatric treatment. We do not have information on whether the psychiatric treatment was initiated, maintained, or discontinued. Third, psychiatric diagnoses of students who died by suicide could not be confirmed and objective assessment of the psychiatric problem was not possible. Fourth, the sample group of our study did not include children and adolescents who dropped out of school. Students in our study may have exhibited fewer mental health problems compared with out-of-school children and adolescents. Fifth, this study does not suggest the effectiveness of psychiatric treatment through school in reducing the suicide rate.

## Conclusion

In conclusion, about approximately 20% of student died by suicide had been advised through their school to seek psychiatric treatment. They showed distinctive clinical characteristics compared to the other 80%. Teachers seem to recommend psychiatric treatment when mental health problems are revealed at schools. Preventing suicide among students requires the attention and effort not only of schools, but also of families, communities, and mental health professionals.

## Data availability statement

The data analyzed in this study is subject to the following licenses/restrictions: This data is from the Korean Ministry of Education and requires permission from the Korean Ministry of Education to access it. Requests to access these datasets should be directed to HH.

## Ethics statement

The studies involving human participants were reviewed and approved by the Institutional Review Board of Hallym University (IRB No: 2021-11-001-001). Written informed consent from the participants’ legal guardian/next of kin was not required to participate in this study in accordance with the national legislation and the institutional requirements.

## Author contributions

HH and HK: conceptualization. HK: formal analysis and writing—original draft. HH: funding. HH and Y-SK: supervision. HH, Y-SK, and HK: writing—review and editing. All authors have read and agreed to the published version of the manuscript.

## Footnotes

1
https://www.who.int/data/gho/data/themes/mental-health

2
https://www.who.int/news-room/fact-sheets/detail/suicide

3
http://kostat.go.kr/portal/korea/kor_nw/1/6/2/index.board?bmode=read&bSeq=&aSeq=403046&pageNo=1&rowNum=10&navCount=10&currPg=&searchInfo=&sTarget=title&sTxt=

4
https://www.moe.go.kr/boardCnts/viewRenew.do?boardID=294&lev=0&statusYN=C&s=moe&m=020402&opType=N&boardSeq=41901

5
https://kfsp.org/sub.php?menukey=10&id=issue&mode=view&idx=264

6
https://www.kdca.go.kr/yhs/home.jsp

7
http://27.101.211.175/mentalhealth/board/boardView.do;jsessionid=3KQoaDMBXBFHguzuoxpOUh7YZBJLbmPahaOoKZo2×1YukKDDpSsHGuzmldx8xCme.mohwwas1_servlet_engine1?no=9011&fno=106&gubun_no=&menu_cd=04_02_00_05&bn=newsView&search_item=&search_content=&pageIndex=1

8
http://27.101.211.175/mentalhealth/board/boardView.do;jsessionid=3KQoaDMBXBFHguzuoxpOUh7YZBJLbmPahaOoKZo2×1YukKDDpSsHGuzmldx8xCme.mohwwas1_servlet_engine1?no=9011&fno=106&gubun_no=&menu_cd=04_02_00_05&bn=newsView&search_item=&search_content=&pageIndex=1

## Abbreviations

SEBT: Students’ Emotional and Behavioral Screening Test
AP: students who were advised to seek psychiatric treatment
NAP: students were not advised to seek psychiatric treatment.
